# Prognostic significance and immune characteristics of CMTM4 in hepatocellular carcinoma

**DOI:** 10.1186/s12885-022-09999-y

**Published:** 2022-08-19

**Authors:** Shengkui Tan, Xuefeng Guo, Chunhua Bei, Huixia Zhang, Di Li, Xiaonian Zhu, Hongzhuan Tan

**Affiliations:** 1 Department of Epidemiology and Statistics, School of Public Health, Guilin Medical University, Guilin, 541199 China; 2 Department of Epidemiology and Statistics, Xiangya School of Public Health, Central South University, Changsha, 410078 China

**Keywords:** Hepatocellular carcinoma, CMTM4, PD-L1, Tumor immunity, CD4, CD8

## Abstract

**Background:**

Previous study has shown that chemokine-like factor (CKLF)-like MARVEL transmembrane domain-containing family member 4 (CMTM4) can bind and maintain programmed cell death ligand 1 (PD-L1) expression to promote tumor progression by alleviating the suppression of tumor-specific T cell activity, suggesting its potential role in tumor immunotherapy. However, the role of CMTM4 in tumor immunity has not been well clarified, especially in hepatocellular carcinoma (HCC).

**Methods:**

The protein expression of CMTM4/PD-L1/CD4/CD8 was detected by immunohistochemistry (IHC) detection in 90 cases of HCC tissues. The mRNA expression profiles and related prognosis data were obtained from The Cancer Genome Atlas-Liver Hepatocellular Carcinoma (TCGA-LIHC). Two immune therapy cohorts were from Imvigor210 and GSE176307.

**Results:**

Though the single protein expression of CMTM4, PD-L1, CD4 or CD8 in HCC tissues by IHC detection didn’t show a significant relationship with the prognosis of HCC patients, we found that high co-expression of CMTM4/PD-L1/CD4 showed a good prognosis of HCC patients. Further Timer 2.0 analysis identified that HCC patients with high expression of CMTM4/PD-L1 and high infiltration of CD4^+^ T cells had a better overall survival than those with low infiltration of CD4^+^ T cells. Moreover, a series of bioinformatics analyses revealed that CMTM4-related genes posed important effects on prognosis and immunity in HCC patients, and CMTM4 had a positive correlation with infiltration of CD4^+^ and CD8^+^ T cells in HCC. At last, we used two immunotherapy cohorts to verify that the combination of CMTM4 with PD-L1 could improve the prognosis of tumor patients underwent immunotherapy.

**Conclusions:**

CMTM4 and PD-L1 co-expression with T cell infiltration shows prognostic significance in HCC, suggesting combined effect from multiple proteins should be considered in HCC treatment.

**Supplementary Information:**

The online version contains supplementary material available at 10.1186/s12885-022-09999-y.

## Introduction

As a common malignant tumor of digestive system, hepatocellular carcinoma (HCC) has a high incidence in sub-Saharan Africa and Asia. According to recent reports, about 55% HCC cases of the world are in China, and the morbidity and mortality of HCC in China reach the second place in all the cancers [1]. Numerous studies have shown that HCC patients could develop micrometastases when they were diagnosed, losing the opportunity to treatments and leading to the poor clinical prognosis [2]. Unfortunately, at present, current treatment is considered extremely difficult to cure HCC metastasis. Over the past decades, immunotherapy and targeted therapies such as CAR-T, immune checkpoint therapy and vaccination, have made significant advances in treating cancer, especially melanoma [3]. However, the main obstacle of immunotherapy is the low response in clinical [4]. Therefore, it is vital to find valuable therapeutic markers for HCC patients.

In recent years, with the deepening understanding of limitations from tumor radiotherapy and chemotherapy, tumor immunotherapy has been paid more attention and clinical application to achieve better results from immune regulation [5]. Programmed cell death 1 (PD-1) and its ligand (PD-L1) have become one of the most studied receptors that brings a recent breakthrough in new tumor immunotherapy for immune checkpoint. PD-L1 expresses in different tumor cells and can directly inhibit the proliferation and function of T cells when combining with PD-1 [6]. Most tumor cells take advantage of PD-1/PD-L1 checkpoint regulation to inhibit immunity and escape immune surveillance [7, 8]. PD-1/PD-L1 signaling pathway is also involved in the process of HCC development which has an impaired immune surveillance function in liver microenvironment [9]. In HCC patients, PD-1 expression was found up-regulated in CD8^+^ T cells [10], and the high abundance of circulating and tumor infiltrating PD-1^+^CD8^+^ T cells was positively correlated with the prognosis of HCC patients under partial hepatectomy and without any immunotherapy before [11]. High expression of PD-1 in tumor infiltrating lymphocytes of HCC patients could cause impaired phenotype and effector function of lymphocytes [12]. Similarly, the expression of PD-L1 in HCC cells can also inhibit the function of T cells in tumor microenvironment. Studies have reported that high PD-L1 expression in tumor cells is an independent prognostic factor for the recurrence of HCC patients [13]. Tissue samples showed the expression level of PD-L1 was high in HCC patients without any immunotherapy before, and had a relationship with the metastasis and poor prognosis of HCC patients [14, 15]. These studies suggest that blocking the interaction between PD-1 and PD-L1 will bring a new dawn for HCC treatment.

Chemokine-like factor (CKLF)-like MARVEL transmembrane domain-containing family (CMTM) members are implicated in autoimmune diseases, male infertility and various tumors [16–18]. Recently, CMTM4 and CMTM6 are identified as PD-L1 regulators to regulate PD-L1 stability during the immune process in many tumor cells [19, 20], and thus expected to be a new target for tumor immunotherapy. Accumulated evidence has been explored to confirm the application potential of CMTM6 in tumor immunotherapy through its interaction with PD-1/PD-L1 signaling pathway [21, 22]. However, the role of CMTM4 in tumor immunity has not been well clarified, especially in HCC.

Our previous study had identified that the deregulated expression of CMTM4 protein was correlated with HCC prognosis [23]. To further explore the prognostic role of CMTM4 in HCC immunotherapy, we did IHC and bioinformatics analysis to detect the co-expression of CMTM4 and PD-L1 in HCC patients, and evaluate the prognostic significance and immune characteristics of CMTM4 and PD-L1 co-expression combined with CD4^+^ or CD8^+^ T cell infiltration. Our study may provide novel immunotherapy clues for HCC patients.

## Methods

### Tissue samples

Our HCC cohort included 90 cases of HCC tissues and paired normal liver tissues from 2005 to 2015. Written informed consent was obtained from each patient. The baseline characteristics of these HCC patients were shown in Table 1. Six PD-L1 samples were excluded due to the loss of immunohistochemical (IHC) sites during IHC quality control. One sample was lost to follow-up in the progression free survival data (*n* = 89). This study was approved by the Institutional Ethical Boards of Guilin Medical University (GLMC2014003).

**Table 1:** The association of clinicopathological parameters with expression of CMTM4 and PD-L1 in HCC cohort

Variables	CMTM4				PD-L1			
	*n*	High	Low	*P* value	*n*	High	Low	*P* value
**Age (years)**^**a**^				0.085				0.880
< 60	68	36	32		63	14	49	
≥ 60	22	7	15		21	5	16	
**Gender**				0.881				0.310
Male	80	38	42		74	18	56	
Female	10	5	5		10	1	9	
**HBV**				0.259				0.473
Positive	70	36	34		66	14	52	
Negative	19	7	12		17	5	12	
**AFP (ng/mL)**				**0.045**				0.456
< 400	57	23	34		54	11	43	
≥ 400	32	20	12		29	8	21	
**Cirrhosis**				0.401				0.578
Yes	80	40	40		74	11	43	
No	9	3	6		9	8	21	
**Cirrhosis number**				0.196				0.477
< 3	56	30	26		51	13	38	
≥ 3	33	13	20		32	6	26	
**Tumor number**				0.261				0.250
Single	79	36	43		73	18	55	
Multiple	11	4	7		11	1	10	
**Tumor size**^**b**^				0.329				0.898
< 5 cm	56	29	27		52	12	40	
≥ 5 cm	34	14	20		32	7	25	
**Differentiation**				0.202				0.112
Well	44	18	26		40	6	34	
Moderate-poor	46	25	21		44	13	31	
**TNM stage**				0.334				0.198
I	63	28	35		61	16	45	
II-IV	27	15	12		23	3	20	
**Tumor capsule**				0.330				**0.040**
Complete	42	18	24		38	15	30	
Incomplete	47	25	22		45	4	34	
**TB (μmol/L)**				0.679				**0.027**
< 14.053	56	28	28		52	16	36	
≥ 14.053	33	15	18		31	3	28	
**ALT (U/L)**				0.996				0.436
< 48.360	60	29	31		55	14	41	
≥ 48.360	29	14	15		28	5	23	
**ALB (g/L)**				0.742				0.077
< 4.296	43	20	23		42	13	29	
≥ 4.296	46	23	23		41	6	35	
**GGT (U/L)**				0.809				0.51
< 77.101	61	30	31		56	14	42	
≥ 77.101	28	13	15		27	5	22	
**CD4 expression**				0.451				0.228
Low	20	6	14		54	10	44	
High	70	37	33		30	9	21	
**CD8 expression**				0.847				**0.001**
Low	21	11	10		41	3	38	
High	69	32	37		43	16	27	

^a^, median age; ^b^, median tumor size;

*P* value < 0.05 in bold is statistically significant

Notes: *AFP* α-fetoprotein, *HBV* Hepatitis B virus infection

### IHC detection

HCC and paired normal liver tissues were made into tissue chips for IHC staining. These tissue chips were incubated by primary antibodies against CMTM4, PD-L1, CD4 and CD8 (anti-CMTM4, ab254657; anti-PD-L1, ab205921; anti-CD4, MABF569; anti-CD8, SAB5500074), respectively at 4 °C overnight. Then the chips were washed three times and incubated with secondary antibodies at 37 °C for 30 min. After stained with 3,3′-diaminobenzidine tetrahydrochloride and hematoxylin, all chips were observed under the Olympus microscope (BX53, Japan).

The IHC scoring was assessed by two professional pathologists independently and they were blinded to the clinicopathological and survival information of patients. Three different stained visual fields in per tissue sample were detected for the percentage of positive cells and the score was estimated by the mean of three percentages. The score in percentage of positive stained cells was defined as 0 for ≤5%, 1 for 6-19%, 2 for 20-49%, 3 for 50-74%, and 4 for 75-100%, respectively. Cell stained intensity was assessed as follows: 0 indicated no staining; 1 indicated weak staining; 2 indicated moderate staining; 3 indicated strong staining. The final scores were obtained by multiplying the percentage of positive cells and the stained intensity. Final score > 3 was defined as positive expression and ≤ 3 was negative expression. The median of four IHC scoring genes was defined as the cut-off value to classify the samples as low or high expression groups, which were determined using previously described methods [24, 25]. Total score (CMTM4/PD-L1, CMTM4/PD-L1/CD4 or CD8) was calculated by adding the every stained intensity score.

### RNA sequencing and data analysis

Total RNA from CMTM4 knockdown (KD) Hep3B cells was extracted using TRIzol (Invitrogen, #15596026). The total RNA concentration and purity were tested by NanoDrop 2000 and Aglient 2100 Bioanalyzer (Thermo Scientific, MA, USA). GeneChip from PrimeView (Affymetrix, #902487) was used for the RNA sequencing and data analysis was provided by GeneChem (Shanghai, China).

### Bioinformatics analysis

#### Data collection

The mRNA expression profiles and related prognosis data were obtained from The Cancer Genome Atlas-Liver Hepatocellular Carcinoma (TCGA-LIHC). Two immune therapy cohorts were included in this study from Imvigor210 and GSE176307. For TCGA-LIHC cohort, RNA sequencing data (fragments per kilobase of exon per million fragments mapped, FPKM) were downloaded (https://portal.gdc.cancer.gov/). 424 samples were included in this cohort. For Imvigor210 cohort, 348 patients treated with the PD-L1 inhibitor (atezolizumab) were used for validation of the combined immunotherapeutic effect of CMTM4 and PD-L1 [26]. 90 patients treated with immune checkpoint inhibition (GSE176307) were used for further analysis in immunotherapy.

#### Classification of CMTM4 related mRNAs

ConsensusClusterPlus is a common tool for identifying the number and membership of clusters for cancer datasets by the K-means method, such as HCC [27, 28]. In order to find significant CMTM4 subgroups, we used ConsensusClusterPlus package (R package) with 50 interactions to get 4 clusters (resample rate of 0.8) according to the differential expressed mRNAs from CMTM4 KD RNA sequencing data. Furthermore, cluster 1-4 was analyzed and visualized by heatmap package and survival package.

#### Identification of CMTM4 related prognostic genes in HCC

In order to construct an efficient CMTM4 prediction model, we used the Least Absolute Shrinkage and Selection Operator (LASSO) Cox regression to select the significant independent prognostic markers with *P* value less than 0.05 for overall survival (OS) in TCGA-LIHC [29]. Then, we got 40 significant prognostic genes. The risk score (RS) of those genes was calculated by the following formula.

$$RS\kern0.5em =\kern0.5em \sum \limits_{i=1}^n Coef(i)X(i)$$

The “n” means 40 genes in this group. Coef (i) represents the coefficient of each gene (Supplementary Table 1). The 365 HCC samples (LIHC) were divided into two groups by the median of risk scores. Then, the prognostic value was analyzed by the Kaplan Meier (KM) method, and sensitively and specificity values were calculated by the receiver operating characteristic (ROC) curve.

#### Relationship between CMTM4 related genes and tumor immunity

To explore the relationship between CMTM4 related differential expressed genes (DEGs) and HCC tumor microenvironment, we used the ESTIMATE (estimation of stromal and immune cells in malignant tumor tissues using expression data) to assess immune score and stromal score for understanding the immune status in HCC patients [30]. Furthermore, Wilcoxon method was used to compare the difference between two immune infiltration scores for clinical information. And to identify the optimal score cutoff in HCC prognostic analyses, “sur.cut” was employed to divide into high and low immune groups by using a “survival” R package. “ggsurplot” function from a R package of “survivmine” was used to draw KM survival curve.

To explore the differences of immune cell subtypes, CIBERSORT package and TIMER2.0 (http://timer.cistrome.org/) were used based on the TCGA-LIHC data [31]. The perm value was set in 1000. The filter condition was set in *P* value < 0.05. Because genes with somatic mutations and somatic copy number variations (sCNVs) could influence immunotherapy response and survival, TIMER2.0 was used to display CMTM4 sCNVs module and related mutation plots for understanding of CMTM4 immune effect. Furthermore, when combined CMTM4 and PD-L1, the outcome module in high and low CD4/CD8 groups was analyzed by CIBERSORT algorithm.

#### CMTM4 related pathways and prognostic genes

To identify important pathways that CMTM4 related genes participate in, we used a “clusterProfiler” R package to analyze Gene Ontology (GO) and KEGG pathway with *P* < 0.05 [32]. Then, the relationship of proteins from CMTM4 significant DEGs were analyzed by STRING (https://string-db.org/). Protein-protein interaction (PPI) analysis was performed by using Cytoscape software. The proteins in the PPI network were transformed into nodes and the degree of each node was related with the number of its interactions. The degree analysis was selected for counting each node and ClusterONE function in Cytoscape was used to screen modules of the PPI network. 32 significant proteins were listed in the barplot (count≥12 and *P* value < 0.05). Cox regression was used to test CMTM4 related DEGs, and 16 proteins of them were important prognostic markers. Then, venn plot was used to find important prognostic markers from PPI. And KM analysis was used to show the prognostic value in those two genes.

#### Immunotherapy analysis

Based on the related studies, the “GSVA” R package was used to quantify the immune infiltration of 28 related cells in HCC microenvironment. ssGSEA classified genes included common biological functions, chromosomal localization, and physiological regulation [33, 34]. Normalized two immunotherapy cohorts (Imvigor210 and GSE176307) data were compared genes by “GSVA” R package. The relative abundance of each immune cell was listed by an enrichment score in ssGSEA analysis. C4P1 (CMTM4 + PD-L1) score was calculated by adding CMTM4 value and PD-L1 value. Based on the mean of C4P1 score, the patients were divided into high and low level groups to assess the immunotherapeutic effect. Patients who got complete response (CR) or partial response (PR) were considered as responders, compared with those non-responders who displayed stable disease (SD) or progressive disease (PD). KM analysis was used to assess the prognostic effect of CD4^+^ T cell in high C4P1 group.

### Statistical analysis

Continuous variables were analyzed using Student’s t-test or Wilcoxon-test. Categorical variables were assessed by Chi-squared test. Patients’ prognostic analyses were showed by KM survival analysis, univariate and multivariate Cox analyses. Survival results were showed using “forestplot” (R package). *P* values < 0.05 was considered as statistically significant. All data were analyzed by R 4.0.5, GraphPad Prism 8 and Cytoscape 3.7.2.

## Results

### Expression of CMTM4/PD-L1/CD4/CD8 in HCC cohort

**Fig. 1:**
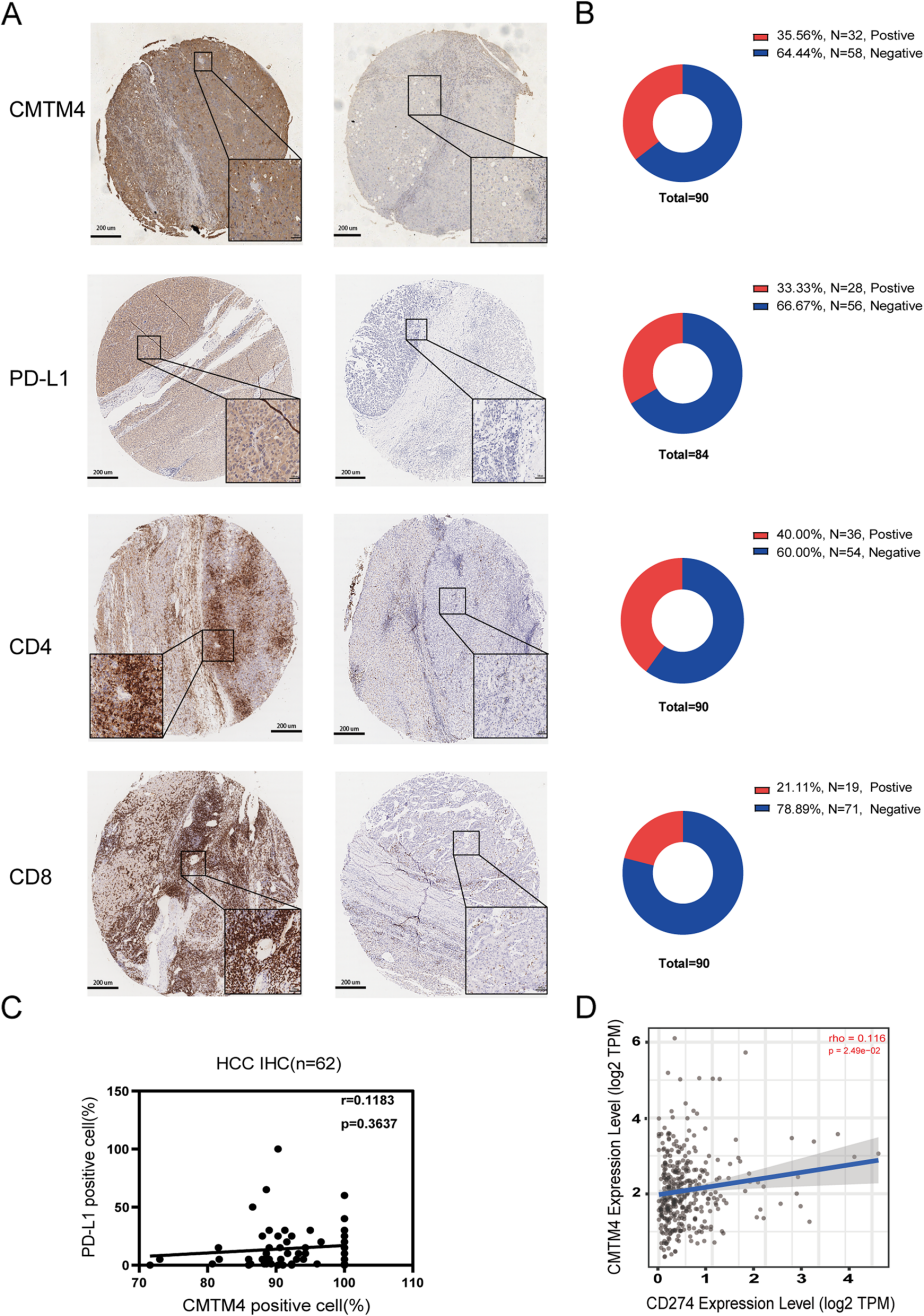
Expression of CMTM4/PD-L1/CD8/CD4 in HCC tissues by IHC detection. **A** Representive positive and negative expression of CMTM4/PD-L1/CD8/CD4 in HCC tissues by IHC detection. **B** Statistical analysis of IHC results in 90 cases of HCC tissues. **C** Correlation between CMTM4 and PD-L1 protein expression from IHC results in HCC cohort. **D** Correlation between CMTM4 and PD-L1 mRNA expression from TCGA-LIHC database

We first detected the expression of CMTM4/PD-L1/CD4/CD8 in a total of 90 HCC tissues from patients who underwent surgical treatment by IHC method. As shown in Fig. 1A, CMTM4, PD-L1, CD4 and CD8 were all expressed in HCC tissues. Based on the IHC score, we found that the positive rate of CMTM4, PD-L1, CD4 and CD8 was 35.56% (32/90), 33.33% (28/84), 40.00% (36/90), and 21.11% (19/90), respectively (Fig. 1B). Then we chose 62 HCC samples that had CMTM4 positive cells above 50% to examine the correlation between CMTM4 and PD-L1 protein expression by Pearson method. As shown in Fig. 1C, there was a same trend between CMTM4 and PD-L1 protein expression in HCC tissues, but the relationship was not significant (r = 0.1183, *P* = 0.3637). The mRNA expression of CMTM4 was found to have a weak positive relationship with PD-L1 in TCGA-LIHC database (rho = 0.116, *P* = 0.00249, Fig. 1D), which was consistent with recent studies in head and neck squamous cell carcinoma and undifferentiated pleomorphic sarcoma [35, 36].

The relationship between CMTM4/PD-L1 expression and clinicopathological characteristics of HCC patients was then analyzed (Table 1). Based on the median of quantitative analysis from IHC, we divided the cohort into high and low groups according to CMTM4 and PD-L1 expression. As a result, we found that high CMTM4 expression was only significantly related with high AFP level (*P* = 0.045), but not was associated with other factors (all *P* > 0.05). High PD-L1 expression was significantly correlated with complete tumor capsule (*P* = 0.040), low total cholesterol level (TB) (*P* = 0.027), and high CD8 level (*P* = 0.001).

### Prognostic significance of CMTM4/PD-L1/CD4/CD8 in HCC cohort

**Fig. 2:**
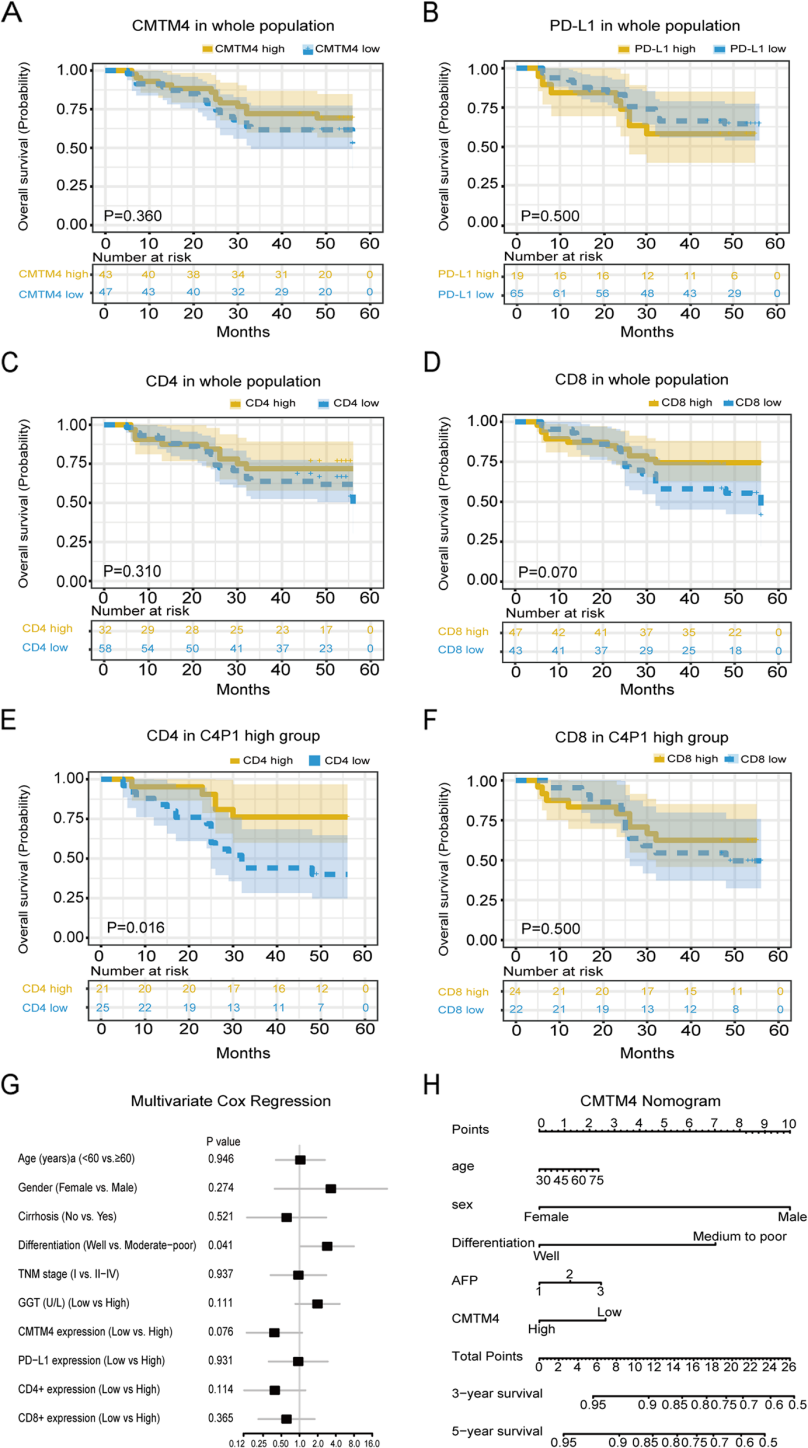
Kaplan-Meier survival curves for overall survival (OS) of HCC patients based on CMTM4/PD-L1/CD4/CD8 protein expression from IHC detection. **A** OS based on CMTM4 expression. **B** OS based on PD-L1 expression. **C** OS based on CD4 expression. **D** OS based on CD8 expression. **E** OS based on the combination of CMTM4, PD-L1 and CD4. **F** OS based on the combination of CMTM4, PD-L1 and CD8. **G** Cox regression analysis of HCC OS factors. **H** Nomogram to predict the 3-year and 5-year OS survival in HCC cohort

To further explore the clinical value of CMTM4/PD-L1/CD4/CD8 in HCC, we conducted KM survival analysis, univariate and multivariate Cox regression to elaborate the prognostic significance of CMTM4/PD-L1/CD4/CD8. KM analysis revealed that only CD8 expression had a significant relationship with the overall survival (OS) of the 90 HCC patients (Fig. 2D, *P* = 0.07). CMTM4, PD-L1 or CD4 expression did not showed meaningful results (Fig. 2A-C, *P* > 0.05). When we further analyzed the co-expression of CMTM4/PD-L1 with CD4 or CD8, we found that high co-expression of CMTM4/PD-L1 with high CD4 expression was significantly related with better OS and progression-free survival (PFS) of HCC patients in a five-year survival period (Fig. 2E, *P* = 0.016; Supplementary Fig. 1E, *P* = 0.062), but high co-expression of CMTM4/PD-L1 with high CD8 expression did not show the significance (Fig. 2F, *P* = 0.50). What’s more, high co-expression of CMTM4/PD-L1 group with high CD4 expression was significantly correlated with better OS of HCC patients in a ten-year survival period (Supplementary Fig. 1G, *P* = 0.022).

In the univariate Cox regression, moderate to poor differentiation, high TNM stage and high glutamine transferase (GGT) level increased the risk of OS (Supplementary Fig. 1H, *P* = 0.01, *P* = 0.021, *P* = 0.018). High CD8 level decreased the risk of OS, but this did not reach significance (*P* = 0.077). After adjusting age, gender and other significant OS factors (cirrhosis, differentiation, TNM stage and GGT), high CMTM4 level decreased the risk of OS and PFS (Fig. 2G, *P* = 0.076). On the contrary, moderate to poor differentiation increased the risk of OS (*P* = 0.041).

At the end, we used nomogram to predict the 3-year and 5-year OS in the HCC cohort (Fig. 2H). Based on the multivariate Cox analysis, we used five significant factors, including differentiation level, CMTM4 expression and common HCC markers (age, gender, AFP) for this prediction. Each subgroup of the five factors corresponded to a point and total point scores were added from each subgroup point. Using the corresponding total point scores, we could predict someone’s 3-year and 5-year prognostic survival rate. For example, one HCC patient aged 60 (points: 1.5), female (points: 0) with well differentiation level (points: 0), had 0-20 ng/ml AFP level (points: 1) and low CMTM4 expression (points: 2.75). The suspected 3-year survival rate of this HCC patient was almost 95% and the 5-year survival rate was 91%.

### CMTM4 related four clusters from consensus clustering and its significant prognostic markers

**Fig. 3:**
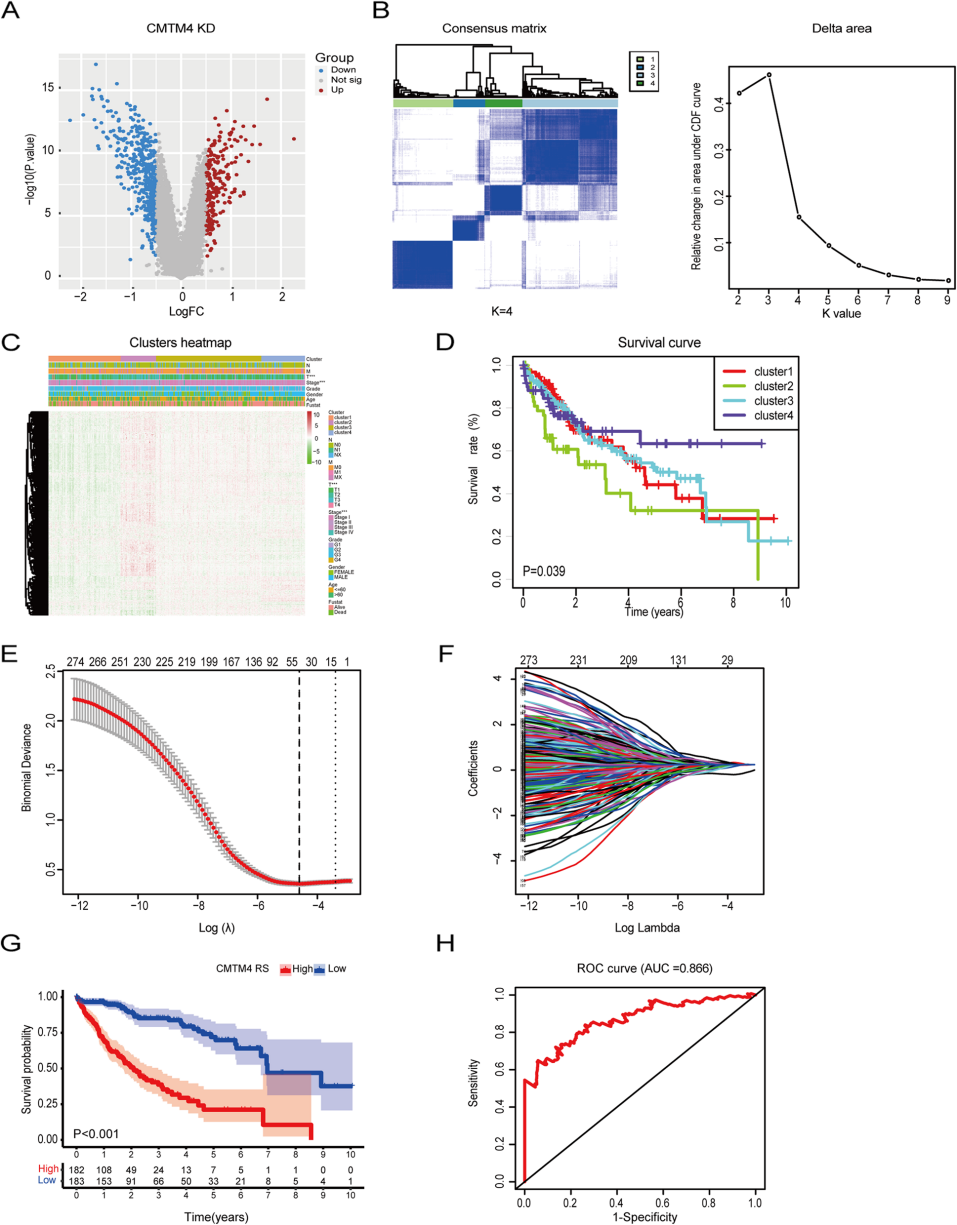
Subgroup analysis of CMTM4-related genes in HCC. **A-E** Consensus clustering analysis of TCGA-LIHC cohort samples at K = 4. **F-I** LASSO Cox regression analysis of TCGA-LIHC cohort samples

In order to investigate the effects of CMTM4 in HCC, RNA-sequencing was performed using CMTM4 KD Hep3B cells. A total of 1189 differentially expressed genes (DEGs) were selected, including 413 up-regulated and 776 down-regulated genes (|log2FC| > 0.5 and *P* value < 0.05, Fig. 3A). Based on the expression of these 1189 DEGs, we clustered the 374 TCGA-LIHC tumor samples to identify CMTM4 prognostic significance using consesus clustering method. The interference between clusters could be reduced to the minimum when K = 3 or 4 (Fig. 3B left). At the same time, when K value is 4, the middle segment of CDF curve is the flattest (Fig. 3B right). Combined with the above two points, we used 4 as K value. Then, the 374 HCC patients were divided into four categories, cluster 1 (*n* = 100), cluster 2 (*n* = 53), cluster 3 (*n* = 159), and cluster 4 (*n* = 62). We then explored the expression of those DEGs among cluster 1-4 and found that there was a lowest level of total gene expression in cluster 1. On the contrary, there was a highest level of overall gene expression in cluster 2. Moreover, when we analyzed the relationship between CMTM4 regulated genes and clinicopathological characteristics of HCC patients, we found that HCC patients in cluster 2 were associated with high T stage and high TNM stage, and instead, HCC patients in cluster 4 were associated with low T stage (Fig. 3D). Further survival analysis indicated that the OS of HCC patients was significantly shorter in cluster 2 than in cluster 4 (Fig. 3D, *P* < 0.05), which may result from its association with high T stage.

We performed univariate Cox regression analysis based on the TCGA-LIHC RNA-sequencing and prognostic data of 374 HCC samples to search valuable CMTM4-related prognostic genes. As a result, 285 genes showed a significant relationship with OS of HCC patients (*P* < 0.05). To identify the most powerful prognostic markers, LASSO Cox regression analysis was used to screen out 21 up-regulated and 19 down-regulated genes (Fig. 3E and F). The coefficient of each gene was showed in Supplementary Table 1. The risk score of the differential expressed 40 genes was calculated based on the coefficient of each marker from LASSO analysis. Based on the median of the risk score, the TCGA-LIHC HCC samples were divided into high and low risk groups. High risk score group (high RS) was shown to have a significant poorer prognosis than the low risk score group (low RS) after the Kaplan-Meier analysis (Fig. 3G). In order to assess the sensitivity and specificity of the prediction, the AUC value was 0.886 from time-dependent ROC curve, suggesting its well prediction ability (Fig. 3H).

### CMTM4 regulates tumor immune environment in HCC

**Fig. 4:**
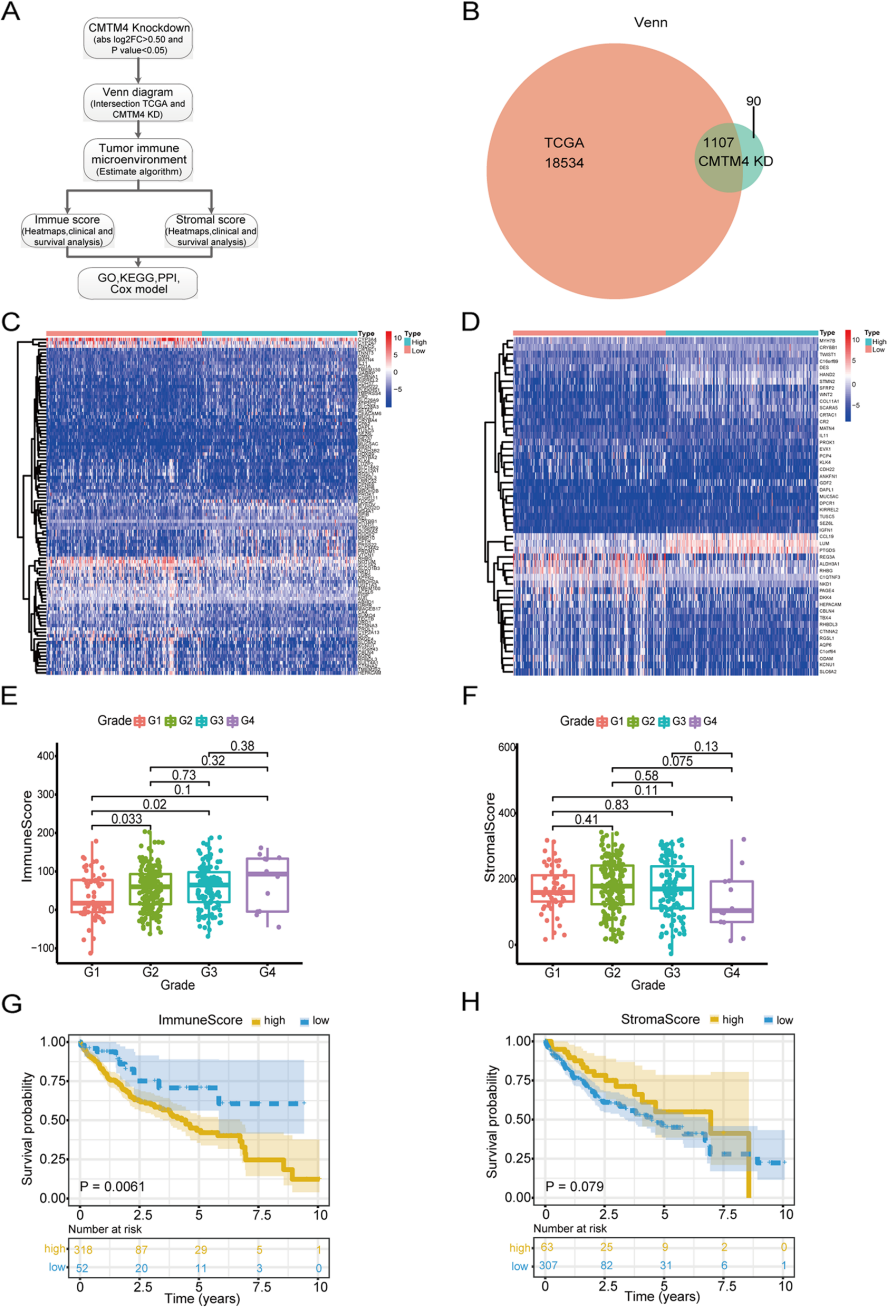
Immune-stromal score estimation of CMTM4-related genes in HCC. **A** Analysis workflow. **B** Venn diagram for the intersection between RNA sequencing data from CMTM4 KD HCC cells and TCGA-LIHC. **C** Immune score estimation. **D** Stromal score estimation. **E** and (**F**) The relationship between immune-stromal score with tumor grade of HCC patients. **G** and (**H**) Correlation of immune-stromal score with OS of HCC patients

As the workflow shown in Fig. 4A, we intersected the RNA sequencing data from CMTM4 KD HCC cells and TCGA-LIHC to assess the exact effect of CMTM4 in tumor microenvironment (TME) of HCC patients. There were 1105 CMTM4-related DEGs included in the HCC TME estimation (Fig. 4B). The top 50 DEGs from immune score included CYP3A4, CYP2A7, and FNDC5, while top 50 DEGs from stormal score included MYH7B, CRYBB1, and TWIST1 (Fig. 4C and D). In order to find relationship between immune score or stormal score and clinical characteristics of HCC patients, we analyzed TNM stage and pathological stage. The results showed that the immune score increased with the tumor grade of HCC patients, especially in grades I-III (Fig. 4E, *P*<0.05). Though stroma score also had an increase trend in grades I-III, it was not significant (Fig. 4F, *P*>0.05). At the same time, KM analysis was used to find its prognostic value from significant TME genes. The results showed HCC patients with high immune score had poor survival compared to low immune score (Fig. 4G, *P* < 0.05), but there was no difference of survival rate between high and low stroma score groups (Fig. 4H, *P* > 0.05).

**Fig. 5:**
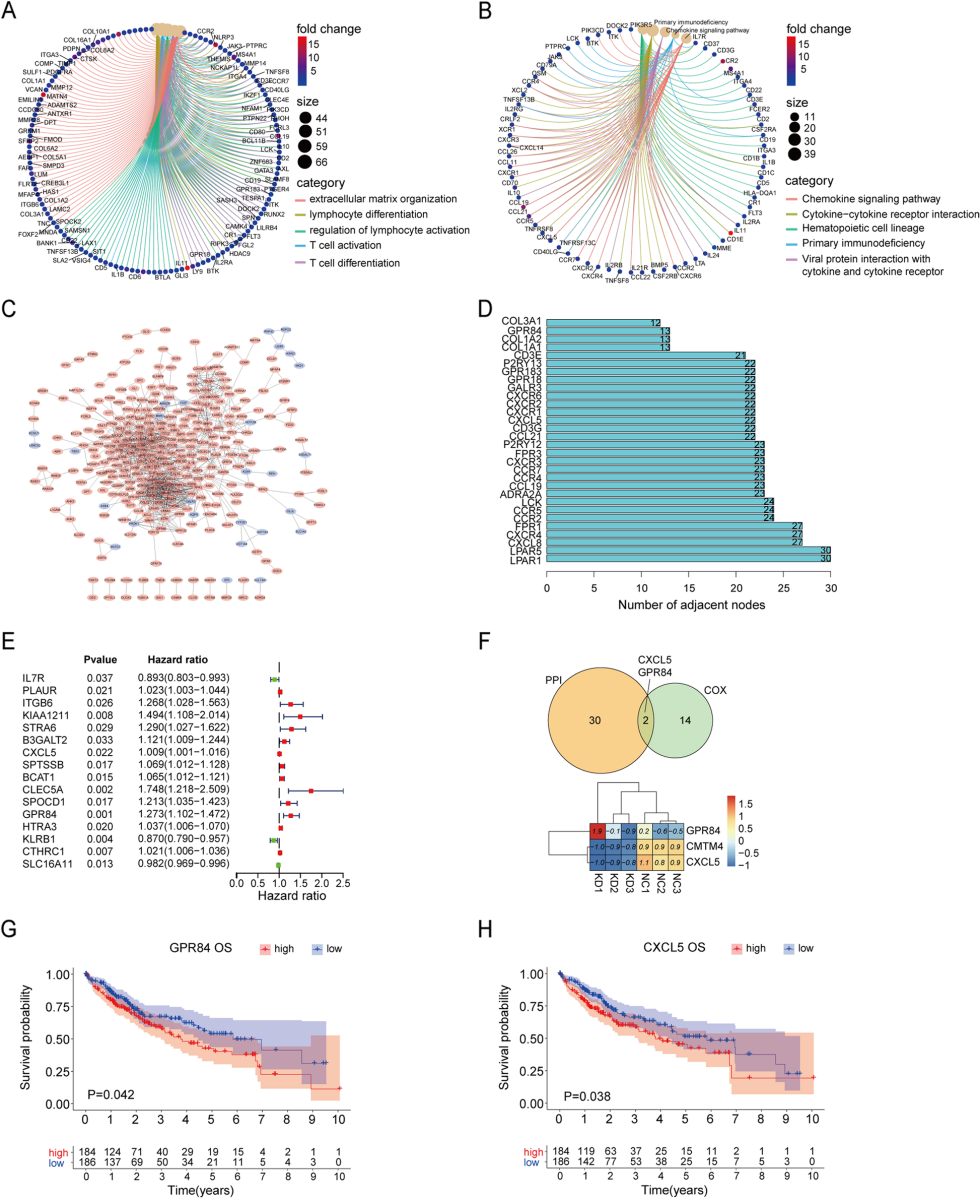
GO, KEGG and PPI analysis of CMTM4-related genes. **A** GO analysis. **B** KEGG analysis. **C** and (**D**) PPI analysis and node number count. **E** Multivariate Cox regression. **F** Venn diagram for the intersection between PPI and Cox results. **G** Correlation of GPR84 with OS of HCC patients. **H** Correlation of CXCL5 with OS of HCC patients

We used GO, KEGG and PPI to analyze the biological processes among CMTM4-related DEGs. As GO result shown in Fig. 5A, these CMTM4-related DEGs were involved in extracellular matrix organization, lymphocyte differentiation, regulation of lymphocyte activation, T cell activation and T cell differentiation. These five pathways included a few same regulated genes, such as CCR2, NLRP3, and JAK3. Chemokine signaling pathway, cytokine-cytokine receptor interaction, hematopoietic cell lineage, primary immunodeficiency and viral protein interaction with cytokine and cytokine receptor were found by KEGG analysis (Fig. 5B). CCR4, TNFSF13B, and CXCR3 were the same regulated genes in the KEGG pathways. Further PPI analysis showed LPAR1, LPAR5, and CXCL5 were the most important proteins, which were involved in regulating cytokines (Fig. 5C). Then top 30 hub genes were enriched in the above significant pathways (Fig. 5D). At the same time, we conducted multivariate Cox regression to further screen the prognostic markers from these CMTM4-related DEGs. As a result, 16 CMTM4-related DEGs were found to have an association with HCC prognosis, including 13 genes with high hazard ratio (HR) and 3 genes with low HR (Fig. 5E). When further intersected these 16 CMTM4-related DEGs with the top 30 hub genes in PPI (Fig. 5D), GPR84 and CXCL5 were screened out (Fig. 5F), which showed both important prognostic value and involvement in regulating the CMTM4-related pathways. In our CMTM4 KD RNA-sequencing data, GPR84 was increased, but CXCL5 was decreased in CMTM4 KD HCC cells. Furthermore, we used Pearson method to find the relationship of CMTM4 with CXCL5 or GPR84. The results showed that CXCL5 and GRP84 were positively related with CMTM4 (Supplementary Fig. 2C and D). Then, Oncomine and four liver cells were used to test CXCL5 and GPR84 expression. The results showed that CXCL5 was up-regulated in HCC cells and tissues, which was consistent with the RNA sequencing result (Supplementary Fig. 2E and G). On the contrary, GPR84 was down-regulated in HCC cells, but was up-regulated in HCC patients (Supplementary Fig. 2F and H). The results of KM analysis showed that HCC patients with high expression of GPR84 or CXCL5 had poorer survival than the low expression group (Fig. 5G and H, *P* < 0.05). These results indicated that CMTM4-related DEGs had both immune and prognostic values in HCC.

### Regulation of immune cell infiltration by CMTM4 in HCC

**Fig. 6:**
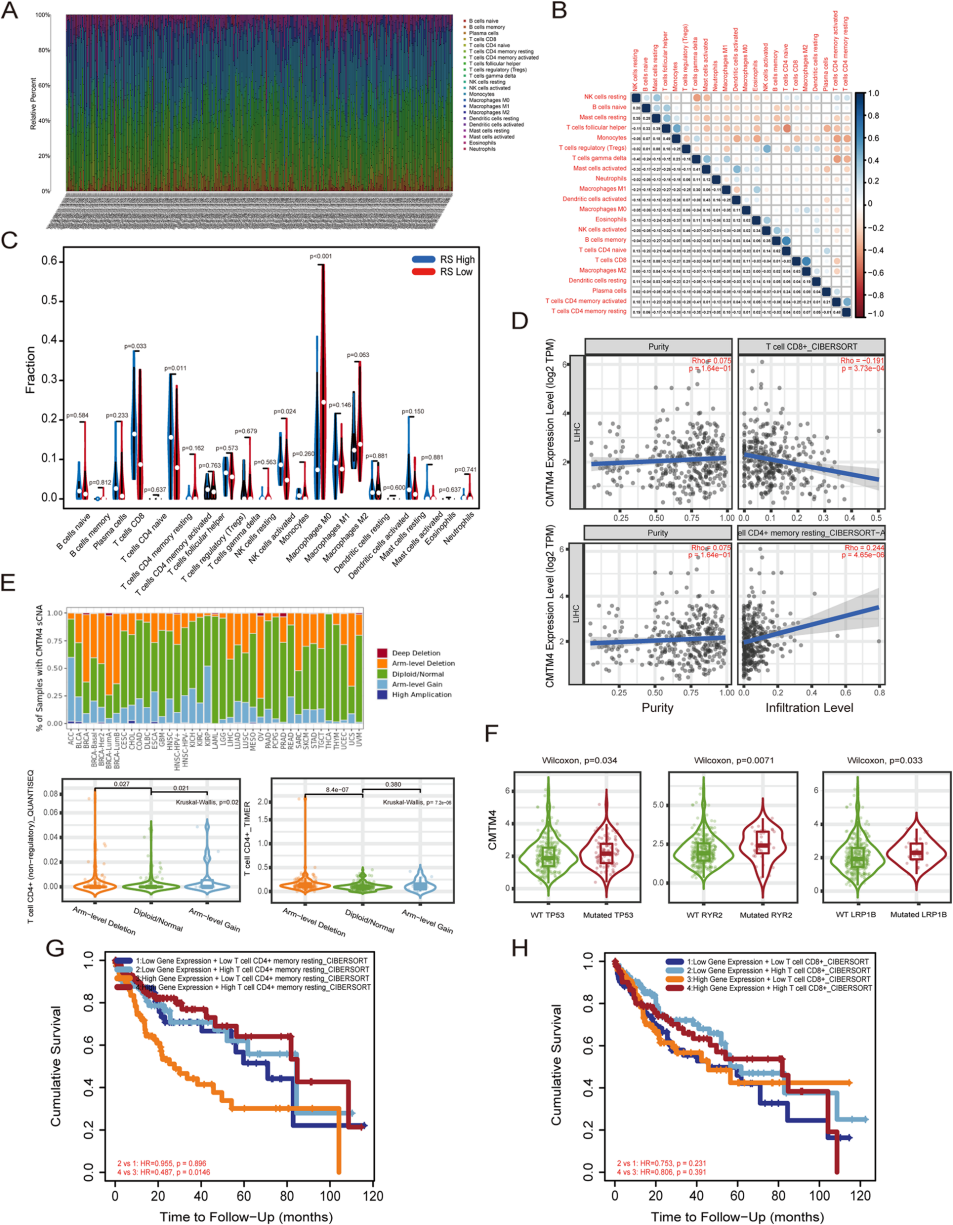
Immune cell infiltration analysis in HCC from TCGA-LIHC database. **A** The proportion of 22 kinds of tumor immune cells (TICs) in HCC. **B** Relationships between the 22 kinds of TICs in HCC. The number in boxes means values of correlation between two kinds of TICs. **C** Violin plots for the ratio differentiation of TICs associated with based on LASSO regression risk score. **D** Relationship between CMTM4 and CD8/CD4 T cells. **E** CNV analysis of CMTM4 gene. **F** Common gene mutation analysis. **G** and (**H**) OS based on the co-expression of CMTM4 and PD-L1 combined with CD4/CD8 expression from TCGA-LIHC database

We analyzed the infiltrating immune cell components in 374 TCGA HCC tumor samples by using CIBERSORT algorithm. The calculation was repeated 100 times and HCC samples with *P* value < 0.05 was chosen for further analysis. As a result, the majority of HCC samples showed four kinds of main immune infiltrating cells, including B cells, T cells, NK cells, and macrophages (Fig. 6A). Further Pearson analysis was employed to find the relationship among the 22 immune cells (Fig. 6B). The results showed that there was a positive correlation between NK cells resting and eosinophils (r = 0.88), T cells CD8 and T cells follicular helper (r = 0.62), T cells CD4 memory activated and neutrophils (r = 0.49), NK cells activated and T cells CD4 native (r = 0.40). In contrast, macrophages M0 were negatively correlated with T cells CD8 (r = − 0.70) and other 8 kinds of immune cells (neutrophils, T cells CD3 memory active, and etc). Macrophage M2 had a negative relationship with Macrophages M1 (r = − 0.43) and other 7 kinds of immune cells. And T cells CD4 memory resting were also negatively related with T cells CD4 memory activated (r = − 0.49) and other 8 kinds of immune cells (Fig. 6B). Then based on CMTM4 risk score (RS) from LASSO regression, high RS group was characterized with elevated infiltration of CD8^+^ T cells, memory resting CD4^+^ T cell, activated NK cells (*P* < 0.05) and decreased infiltration of M0 macrophages (*P* < 0.001, Fig. 6C). Further TIMER2.0 predicted that CMTM4 had a negative correlation with CD8^+^ T cells (Rho = − 0.191, *P* < 0.05), but a positive correlation with CD4^+^ T cells (Rho = 0.244, *P* < 0.05, Fig. 6D). CNV analysis found that arm-level deletion and arm-level gain alterations of CMTM4 mRNA affected immune genes, especially CD4^+^ T cell expression (Fig. 6E), suggesting a close relationship between CMTM4 and CD4^+^ T cell expression in HCC. Meanwhile, analysis of common gene mutations also revealed that TP53, RYR2, LRP18 gene mutations can affect CMTM4 expression (Fig. 6F). Prognostic analysis confirmed that patients with high levels of CD4^+^ T cells in the high CMTM4 and PD-L1 group (high C4P1 group) had good prognostic survival (Fig. 6G). In contrast, this was not found in CD8^+^ T cells (Fig. 6H). These results showed that the effect of CMTM4 on immune cell infiltration posed prognostic significance in HCC.

### Roles of CMTM4 in tumor cohorts underwent immunotherapy

**Fig. 7:**
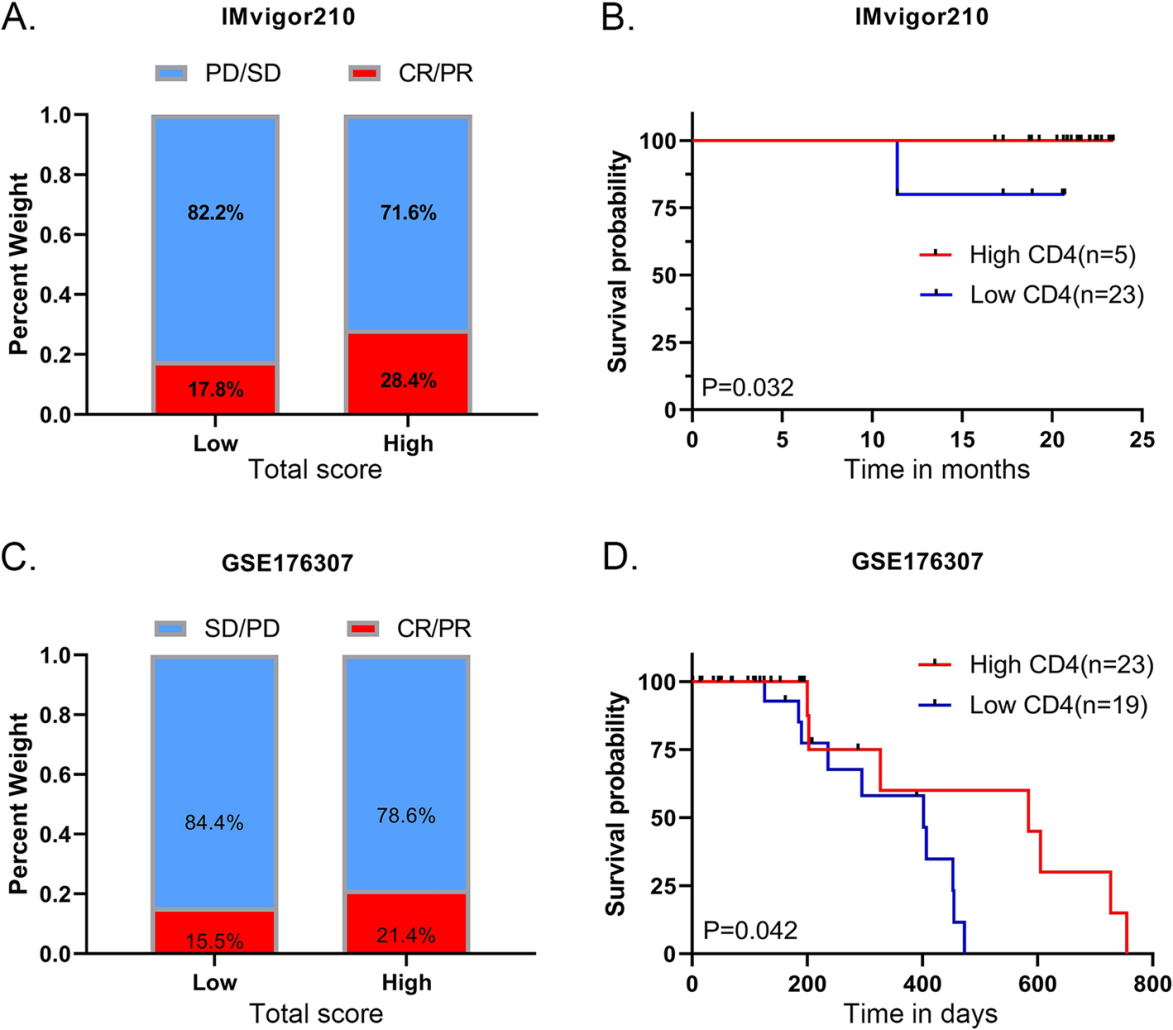
Roles of CMTM4 in tumor cohorts underwent immunotherapy. **A** and **B** IMvigor immune cohort. **C** and (**D**) GSE176307 cohort

Because of the important effects of CMTM4-related genes on prognosis and immunity in HCC patients, we used two immunotherapy cohorts to verify whether the combination of CMTM4 with PD-L1 could improve the prognosis of tumor patients underwent immunotherapy. The IMvigor immune cohort found that immunotherapy patients with high total score had higher immunotherapy correspondence as compared to the low total score group (high 28.4% vs low 17.8%, Fig. 7A). Consistent results were found in another immunotherapy cohort (GSE176307) (high 21.4% vs low 15.5%, Fig. 7C). Furthermore, in both immune cohorts, the results of KM prognostic analysis showed that patients with high total score did have better survival when combined with high CD4 infiltration compared to low CD4 infiltration (Fig. 7B and D, *P* < 0.05), suggesting that CMTM4 combined with PD-L1 could affect CD4^+^ T cell immune infiltration to improve the immunotherapy effect and prognosis of tumor patients.

## Discussion

In this study, we firstly explored the prognostic significance of CMTM4 and PD-L1 co-expression in HCC combined with CD4 and CD8 T cell infiltration data both from IHC detection and TCGA database. High co-expression of CMTM4/PD-L1 with high CD4 T cell immune infiltration was found to show a favorable prognosis of HCC patients. Our study provides a promising role of CMTM4 in HCC immunotherapy and a further theoretical foundation for the application of PD-1/PD-L1 inhibitors in HCC treatment.

As one of the key regulators of PD-L1, the potential role of CMTM6 in tumors has been extensively studied in recent years [37–39]. Its closet family member CMTM4 shares this function that maintaining PD-L1 protein expression [20]. Ectopic expression of circular RNA CDR1-AS can both increase expression levels of cell surface PD-L1, and CMTM4/CMTM6 in SW620 cells [40]. Due to high expression of PD-1/PD-L1 in tumor-infiltrating T cells, CMTM4 and CMTM6 are thought to be important players in the tumor microenvironment and anti-tumor immunity [41]. However, there are only a few studies to clarify the relationship between CMTM4 and tumor immunity. CMTM4 expression was found to have a positive correlation with CD8^+^ and PD-1^+^ cell density in the stroma of head and neck squamous cell carcinoma [35]. Three immune molecular subtypes of renal clear cell carcinoma based on CTLA-4, PD-1/PD-L1, CMTM6 and CMTM4 were found with different clinical and immunological characteristics [42]. CMTM4 can stabilize the surface expression of PD-L1 in HCC and intrahepatic cholangiocarcinoma (ICC) cell lines through preventing PD-L1 from endosome-lysosomal and proteasomal degradation [43]. In this study, though we failed to find the significance of single CMTM4, PD-L1, CD4 or CD8 expression in the prognosis of HCC patients due to small HCC sample size for IHC detection, the high co-expression of CMTM4 and PD-L1 combining with high CD4 expression showed a better HCC prognosis, further supporting that CMTM4 as a regulatory factor in HCC TME could control the immune effect of PD-L1. Therefore, the interaction between CMTM4 and PD-L1 made a significant impact on tumor microenvironment through targeting CD4 T cells, and then promoted the malignant progression of HCC.

HCC development is often accompanied by the accumulation of immune tolerance signals and immune escape. PD-1/PD-L1 is an important inhibitory pathway that involved in immune escape of various tumors through regulating T lymphocytes, which play an important role in the tumor-specific immune response. On the locally infiltrated effector cells in HCC tissues, the increase of PD-1/PD-L1 expression level was found positively correlated with AFP level, vascular invasion, differentiation degree and histological type of HCC patients [14]. Researchers over-expressed PD-1 plasmids in animal HCC tissues and confirmed that PD-1 could enhance the immune response. High expression level of PD-1/PD-L1 in cells could inhibit the effector cells to make HCC immune escape. Moreover, they found that the progression of HCC can be controlled after blocking PD-1/PD-L1 pathway [44]. In consistent with these studies, our immune cell infiltration results also showed that CMTM4 expression had a positive relationship with CD4 and CD8 T cell infiltration levels, and co-expression of CMTM4/PD-L1 combined with CD4 T cell infiltration posed a positive effect on HCC prognosis, suggesting CMTM4 as a trigger combined with PD-L1 could improve tumor microenvironment of HCC by targeting T lymphocyte infiltration.

As the dominant immune response in anti-tumor immunity, cellular immune response is mainly composed by T lymphocytes, especially CD4 and CD8 regulatory T lymphocytes. The CD4 and CD8 regulatory T lymphocytes are immune cells with negative regulating role in anti-tumor immunity through participating in tumor cell escape, immune surveillance and defense [45, 46]. Studies showed that the intratumoral infiltration of CD4 and CD8 T lymphocytes was associated with postoperative recurrence rate and overall survival rate of colorectal cancer patients [47], and patients with a high infiltration of CD8 T lymphocytes in HCC tissues had a better prognosis [48]. Moreover, Langhans B et al. found that there were a higher proportion of CD4 and CD8 T lymphocytes in the peripheral blood of HCC patients than the healthy controls [49]. Zhong et al. confirmed that the overall survival and tumor-free survival rate of HCC patients with high infiltration of CD4 and CD8 T lymphocytes were significantly higher than those in low infiltration group, and their multivariate analysis showed that high infiltration of CD4 and CD8 T lymphocytes was a protective factor for HCC tumor recurrence and prognosis [50]. We found a favorable prognosis of HCC patients with high CMTM4/PD-L1 expression and high CD4 T lymphocyte infiltration in HCC tissues, which was consistent with above studies. Moreover, our bioinformatics analysis also identified that CMTM4-related DEGs were involved in HCC immunity regulation and showed prognostic value in HCC, further providing a possible theoretical evidence for the prognostic significance of CMTM4 and PD-L1 co-expression combined with T cell infiltration in HCC.

As an immune-based therapy, immune checkpoint inhibitors (ICIs) are shown to have a systemic treatment for malignant tumors [51, 52]. ICIs such as PD-1/PD-L1 or cytotoxic T-lymphocyte (CTLA-4) can produce robust and durable response in several cancer patients, including advanced HCC [53, 54]. However, only a minority of HCC patients obtained benefit from single ICI treatment. Researchers believed that HCC immunotherapies combined with standard therapies would exert a higher effect according to the clinical trials for ICIs in HCC treatment [55]. Recent studies showed that ICI combination therapy and co-expression of two genes provided a new strategy for treatment of HCC. In IMBRAVE150 trial, HCC patients who treated with the combination of PD-L1 inhibitor with VEGF inhibitor had better survival than patients who treated with single sorafenib [56, 57]. Luo et al. observed that co-expression of IL7 and CCL21 could enhance the effect of CAR-T cells in solid tumors [58]. And then Peng et al. also showed similar results that patients with high co-expression status of CMTM6 and PD-L1 had the longest survival, suggesting CD4/CD8 T cells provided an active immune microenvironment [59]. Notably, consistent with above studies, we also demonstrated that co-expression of high CMTM4 and PD-L1 with high CD4 had better prognosis in two PD-L1/PD-1 treatment cohorts. These results suggested the HCC patients with co-expression of these genes might boost immune response of immune cells in the HCC microenvironment.

Because of the small sample size and the different sample source from previous studies, single protein expression of CMTM4, PD-L1, CD4 or CD8 in HCC tissues by IHC detection didn’t show a significant correlation with the prognosis of HCC patients. The exact mechanism for the co-expression of CMTM4/PD-L1/CD4/CD8 in HCC tissues needs to be elaborated in a larger HCC cohort from multiple sources. In addition, the prognostic significance of CMTM4/PD-L1/CD4/CD8 co-expression in HCC indicates that the effect of a single protein in HCC microenvironment is relatively weak, HCC progression is promoted by the combination and cooperation from multiple proteins. Our study also explains the current difficulty in HCC treatment, which may be improved through the combination of various treatments.

## Supplementary Information

**Additional file 1.**

**Additional file 2.**

**Additional file 3.**

## Data Availability

Publicly available datasets were analyzed in this study. The RNA sequencing data from CMTM4 knockdown (KD) Hep3B cells can be found here: https://www.ncbi.nlm.nih.gov/geo/query/acc.cgi?acc=GSE186815.
