# Supplementary material for: Prognostic significance and immune characteristics of CMTM4 in hepatocellular carcinoma
Source: BMC Cancer. 2022 Aug 19;22:905. doi: 10.1186/s12885-022-09999-y (PMC9389844; doi:10.1186/s12885-022-09999-y)
Supplement: Supplementary file 1 — Additional file 1. [file 12885_2022_9999_MOESM1_ESM.docx]

**Supplementary Table 1.** Coefficient of each gene in LASSO analysis

| **Gene** | **Coef** |
| --- | --- |
| Intercept | -1.13761 |
| RPUSD4 | 0.24036 |
| EIF2S1 | 0.115079 |
| CHODL | 0.108321 |
| KIFAP3 | 0.097089 |
| ARRDC3 | 0.091725 |
| ABLIM3 | 0.090365 |
| GAL | 0.08982 |
| HSD17B12 | 0.089011 |
| MAP7D3 | 0.071789 |
| AKIP1 | 0.071388 |
| ARL5B | 0.070634 |
| MYCN | 0.069693 |
| AKIRIN1 | 0.062682 |
| KRT17 | 0.06264 |
| SLC7A11 | 0.056698 |
| LMO4 | 0.034748 |
| RRAGC | 0.027411 |
| IQGAP3 | 0.018291 |
| ZIC2 | 0.014292 |
| POMZP3 | 0.002093 |
| LOX | 0.000677 |
| SLC2A2 | -0.00628 |
| CFH | -0.01618 |
| PIK3R1 | -0.01764 |
| TXNIP | -0.02152 |
| BAIAP2 | -0.02349 |
| GRAMD1C | -0.02445 |
| SERPINA3 | -0.02683 |
| ADH4 | -0.02996 |
| SRSF12 | -0.04585 |
| BDH1 | -0.04886 |
| NOSTRIN | -0.06884 |
| KCNK1 | -0.08354 |
| CGNL1 | -0.08972 |
| RAPGEF2 | -0.12222 |
| FRRS1L | -0.14676 |
| SOCS2 | -0.15012 |
| SYTL5 | -0.15892 |
| KLF2 | -0.20066 |
| ALG5 | -0.27045 |

**Supplementary Figures**

**Supplementary Figure 1. K**aplan-Meier survival curves for progression-free survival (PFS) of HCC patients based on CMTM4/PD-L1/CD4/CD8 protein expression from IHC detection. (**A**) PFS based on CMTM4 expression. (**B**) PFS based on PD-L1 expression. (**C**) PFS based on CD4 expression. (**D**) PFS based on CD8 expression. (**E**) PFS based on the combination of CMTM4, PD-L1 and CD4. (**F**) PFS based on the combination of CMTM4, PD-L1 and CD8. (**G**) OS in ten years based on the combination of CMTM4, PD-L1 and CD4. (**H**) Univariate Cox regression analysis of HCC OS factors.

**Supplementary Figure 2.** Expression of CMTM4/CXCL5/GPR84 in HCC samples and cells. (**A**) CMTM4 expression in TCGA-LIHC. (**B**) CMTM4 expression after knocking-down in Hep3B cells. (**C**) Relationship between CMTM4 and CXCL5 expression in TCGA-LIHC. (**D**) Relationship between CMTM4 and GPR84 expression in TCGA-LIHC. (**E**) CXCL5 expression in GSE14520. (**F**) GPR84 expression in TCGA-LIHC. (**G**) CXCL5 expression in HCC cells. (**H**) GPR84 expression in HCC cells.
