# Supplementary figures and images for: Prognostic significance and immune characteristics of CMTM4 in hepatocellular carcinoma
Source: BMC Cancer. 2022 Aug 19;22:905. doi: 10.1186/s12885-022-09999-y (PMC9389844; doi:10.1186/s12885-022-09999-y)

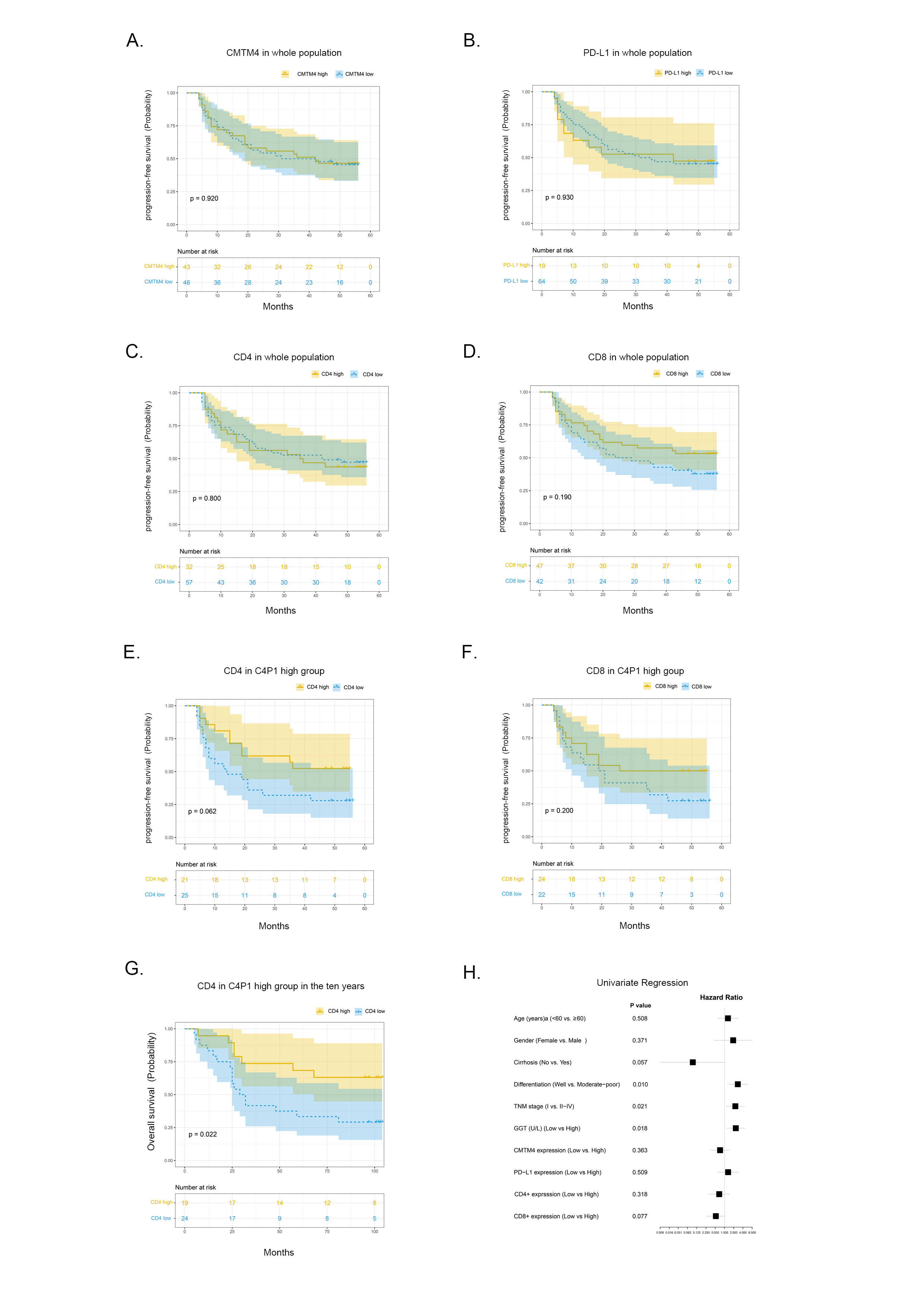

Supplement: Supplementary file 2 — Additional file 2. [file 12885_2022_9999_MOESM2_ESM.jpg]

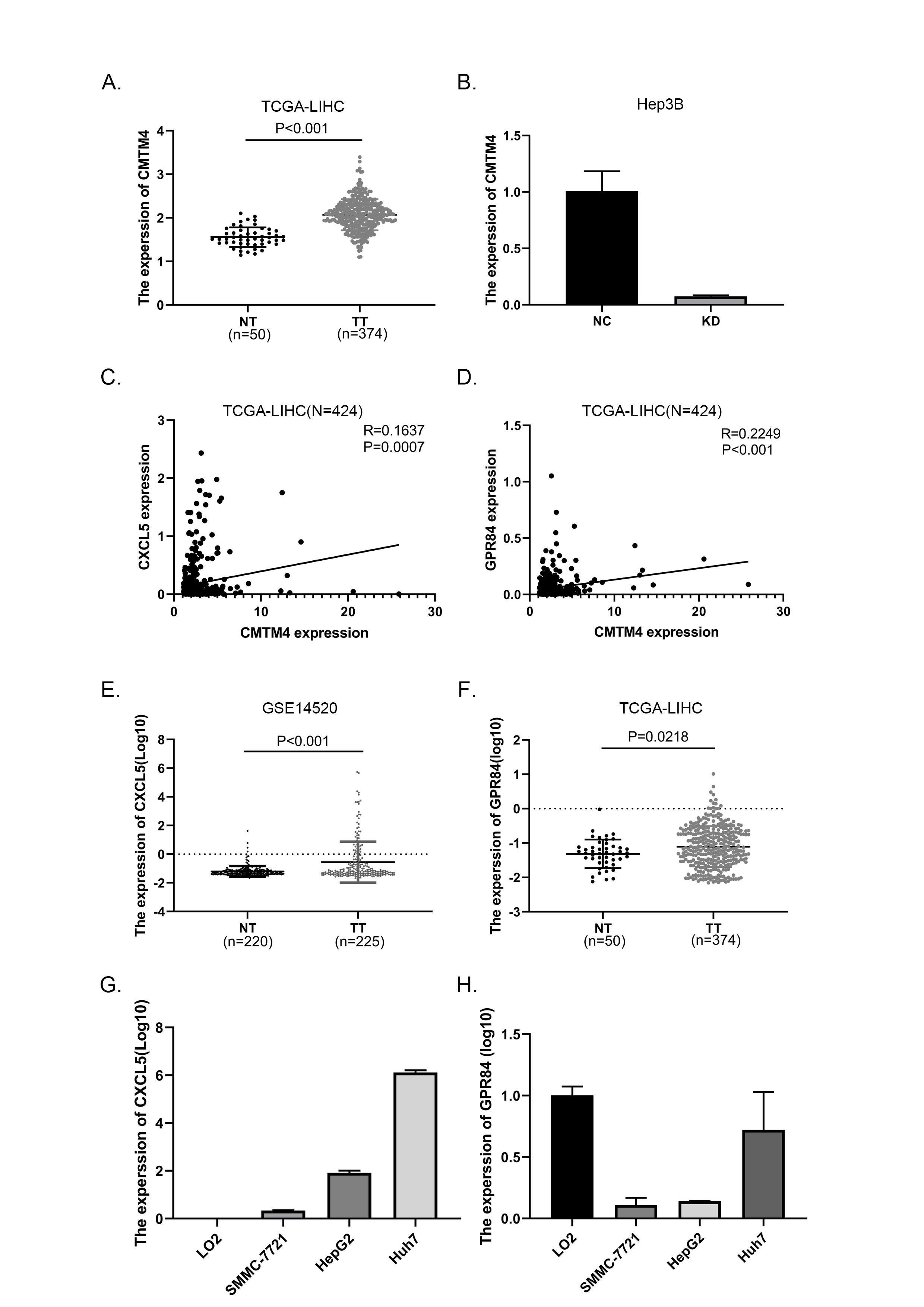

Supplement: Supplementary file 3 — Additional file 3. [file 12885_2022_9999_MOESM3_ESM.jpg]
